# The Role of Female and Male Genes in Regulating Pollen Tube Guidance in Flowering Plants

**DOI:** 10.3390/genes15111367

**Published:** 2024-10-24

**Authors:** Siyuan Zheng, Feng Wang, Zehui Liu, Hongbin Zhang, Liangsheng Zhang, Dan Chen

**Affiliations:** 1 Hainan Institute, Zhejiang University, Sanya 572025, China; 22216132@zju.edu.cn (S.Z.); 22216157@zju.edu.cn (F.W.); 18435221586@163.com (Z.L.); 2 Zhejiang Provincial Key Laboratory of Horticultural Plant Integrative Biology, College of Agriculture and Biotechnology, Zhejiang University, Hangzhou 310058, China; zls83@zju.edu.cn; 3 Sanya Nanfan Research Institute, Hainan University, Sanya 572025, China; 23220951310066@hainanu.edu.cn; 4 Yazhouwan National Laboratory, Sanya 572025, China

**Keywords:** pollen tube guidance, female gametophyte, polytubey block, polyspermy

## Abstract

In flowering plants, fertilization is a complex process governed by precise communication between the male and female gametophytes. This review focuses on the roles of various female gametophyte cells—synergid, central, and egg cells—in facilitating pollen tube guidance and ensuring successful fertilization. Synergid cells play a crucial role in attracting the pollen tube, while the central cell influences the direction of pollen tube growth, and the egg cell is responsible for preventing polyspermy, ensuring correct fertilization. The review also examines the role of the pollen tube in this communication, highlighting the mechanisms involved in its growth regulation, including the importance of pollen tube receptors, signal transduction pathways, cell wall dynamics, and ion homeostasis. The Ca^2+^ concentration gradient is identified as a key factor in guiding pollen tube growth toward the ovule. Moreover, the review briefly compares these communication processes in angiosperms with those in non-flowering plants, such as mosses, ferns, and early gymnosperms, providing evolutionary insights into gametophytic signaling. Overall, this review synthesizes the current understanding of male–female gametophyte interactions and outlines future directions for research in plant reproductive biology.

## 1. Introduction

In mosses, ferns, and some gymnosperms, sperm cells possess motile flagella that enable them to swim to the egg cell for fertilization. In contrast, gymnosperms exhibit the first appearance of pollen tube structures, a mechanism that has been extensively studied in angiosperms. In flowering plants (angiosperms), fertilization is predominantly facilitated by the pollen tube, which is subject to selective pressure; the first pollen tube to reach the ovule’s micropyle gains entry and completes fertilization.

Within angiosperms, ovules are situated within specialized carpels that collectively form a conduit. In angiosperms, using *Arabidopsis thaliana* as an example, after landing on the stigma, the pollen grain germinates and develops a pollen tube. The pollen tube grows through the stigma towards the ovule, carrying the two sperm cells. During this process, it receives certain signals secreted by the female gametophyte (embryo sac, composed of synergid cells, an egg cell, a central cell, and antipodal cells), guiding its growth towards the embryo sac. The guidance of the pollen tube is a highly regulated process involving mutual recognition between the female gametophyte and the male gametophyte cells [1]. This journey includes two critical stages: funicular guidance and micropylar guidance [2].

After pollen grains land on the stigma of the pistil, germination of a pollen tube transports the two sperm cells and a vegetative cell nucleus through the stigma to the transmitting tract. As the pollen tube exits the transmitting tract, it responds to attractant signals, extends along the surface of the funiculus, and proceeds towards the micropyle. This journey includes two critical stages: funicular guidance and micropylar guidance [2]. The guidance of the pollen tube is a highly regulated process involving mutual recognition between the female gametophyte (embryo sac, composed of synergid cells, an egg cell, a central cell, and antipodal cells) and the male gametophyte cells [1]. This coordination governs the rapid growth of pollen tubes within the pistil and ensures accurate targeting of the ovules. Such interactions are essential for signal exchange and effective engagement between female and male gametophytes.

Within angiosperms, ovules are situated within specialized carpels that collectively form a conduit. During their transit through the transmitting tract, pollen tubes navigate a complex path before reaching the ovule. They first penetrate the micropyle and subsequently release sperm cells at the designated site, completing the process of double fertilization. This approach minimizes pre-fertilization investment, amplifies competition, and enhances the adaptability of angiosperms to their environment.

The discovery of double fertilization in angiosperms is relatively recent, dating back just over a century. Significant advancements have been made in the past two decades. The embryo sac within angiosperm ovules is highly specialized, and most components of the female gametophyte (synergid cell, egg cell, central cell) contribute to attracting the pollen tube. Extensive reviews have covered the interactions between the stigma and pollen [3,4,5,6], including the signaling systems required for pollen tube navigation, the potential roles of Ca^2+^ signals [7,8,9,10,11,12], the development of the female gametophyte [13,14,15], and the evolution of the central cell [16].

This review summarizes recent research on the roles of female gametophyte cells and the pollen tube in pollen tube guidance and references studies on sperm cell guidance in archegoniate plants.

## 2. The Role of Female Gametophyte Cells in Male–Female Communication in Flowering Plants

In flowering plants, the female gametophyte consists of synergid cells, an egg cell, and a central cell, with some species also having antipodal cells. Synergid cells play a crucial role in attracting the pollen tube by releasing small peptides. When the synergid-dependent attraction system fails, the central cell can rescue pollen tube guidance by secreting attractant peptides. Additionally, the egg cell also participates in guiding the pollen tube. Table 1 provides a summary of the genes involved in pollen tube guidance that are expressed across all components of the female gametophyte (Table 1).

### 2.1. Synergid Cells in Pollen Tube Attraction

During the process of the ovule attracting the pollen tube, the synergid cells play the primary role in attraction. The ovule of *Arabidopsis* has a Polygonum-type embryo sac, containing two synergid cells. When the pollen tube reaches the embryo sac, it ruptures at one synergid cell, releasing two sperm cells (the synergid cell also ruptures simultaneously). These sperm cells then fertilize the egg cell and the central cell. Induced by central cell fertilization, the persistent synergid cell fuses with the endosperm, which dilutes synergid cytoplasm with pollen tube attractants to prevent polytubey [20,22]. If there is an obstacle during the double fertilization process, preventing its normal completion, the persistent synergid cell can attract another pollen tube, thereby rescuing fertilization.

In 2001, Higashiyama conducted an experiment using a laser to ablate female gamete cells in the embryo sac of *Torenia fournieri* [63]. The guidance of pollen tubes in ovules where both synergid cells were ablated was completely lost, whereas it was affected to varying degrees in other cases [63]. This confirmed the predominant role of synergid cells in attracting pollen tubes [63].

**Figure 1:**
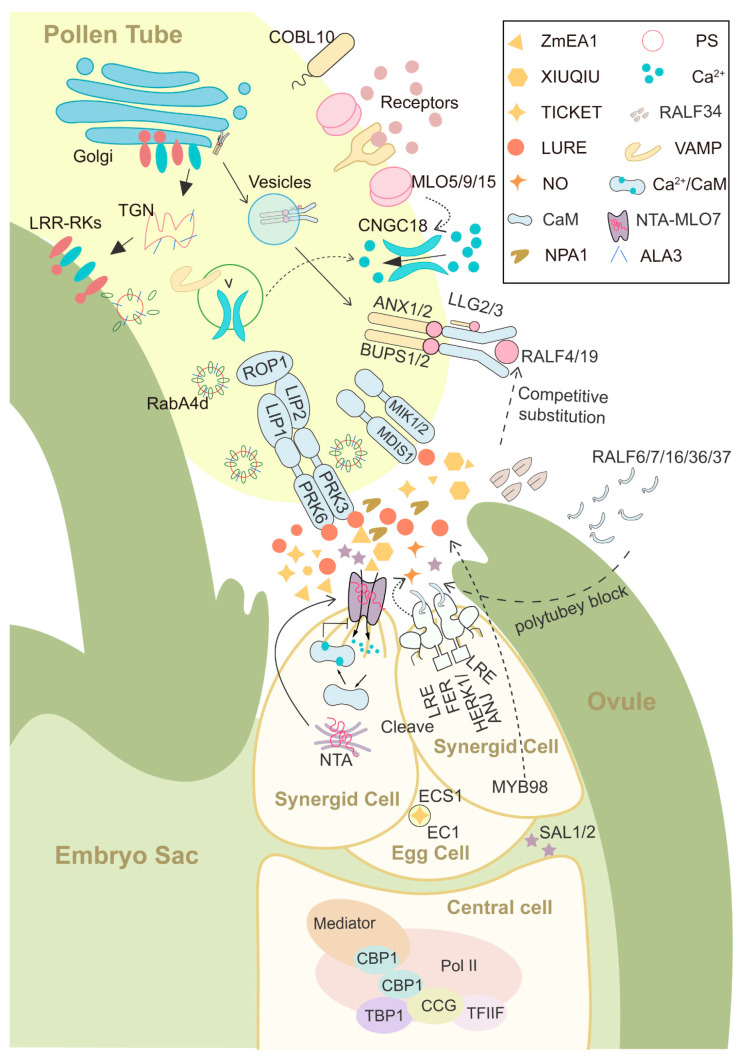
The pollen tube is targeting the ovule. LUREs, TICKETs, and XIUQIUs secreted by synergids are attractants that attract pollen tubes. LUREs specifically bind to PRK6 and MDIS1-MIK1/2 at the tip of pollen tubes. FER interacts with LRE, HERK1, and ANJ to recruit NTA from the Golgi to the filiform apparatus and form a Ca^2+^ channel, mediating pollen tube rupture. ECS1 secreted by egg cells can cleave LURE to avoid polyspermy. The *CCG* located in the central cell and its binding protein CBP1 are also involved in pollen tube guidance. SAL1/2 is secreted into the micropyle after fertilization failure to restore fertilization ability. The MLO at the tip of the pollen tube recruits the Ca^2+^ channel CNGC18 to the membrane. At the same time, when the pollen tube reaches the micropyle, the peptide ligand RALF4/19 of ANX-BUPS is competitively replaced by RALF34, inducing the rupture of the pollen tube. RALF6/7/16/36/37 binds to the FER-ANJ-HERK1 complex to establish a polytubey block.

The key transcription factor *MYB98* (Figure 1) specifically regulates the generation of synergid cells. The morphology of synergid cells in *myb98* mutants remained relatively unaffected, but pollen tube guidance and filiform apparatus (a finger-like protrusion structure located at the micropyle end of the synergid cell) formation were defective [30]. *MYB98* is localized in the nucleus of synergid cells, regulating multiple downstream CRPs (cysteine-rich proteins) [31,32].

Small cysteine-rich defensin-like polypeptides were found to be the attractants expressed by synergid cells [64]. In *T. fournieri*, two CRPs, *Tf*LURE1 and *Tf*LURE3, are abundantly expressed in synergid cells and are secreted to the surface of the egg apparatus (egg cell and synergid cells). These peptides showed activity in vitro, attracting competent pollen tubes of their own species [33]. A methyl-glucuronosyl arabinogalactan (AMOR) is abundant in the ovary and helps induce pollen tube competency to respond to the LURE signal [34,51,65]. Subsequently, several small peptides were discovered in *Arabidopsis* that are specifically expressed in synergid cells and released at the micropyle, playing a role in pollen tube guidance, such as *At*LURE1, XIUQIUs, TICKETs, and the newly discovered NPA1 (NON-DEFENSIN PEPTIDE ATTRACTANT 1) (Figure 1) [35,36,37,53]. They are all CRPs except for NPA1. Among these, only XIUQIUs exhibits a similar level of attraction between *A. thaliana* and its relative species.

After the pollen tube reaches the ovule, its successful rupture to release sperm cells is regulated by several genes. Members of the *Cr*RLK1L (*Catharanthus roseus* receptor-like kinase 1-like) family and their peptide ligand RALF (Rapid Alkalinization Factor) play important roles in this process, including FERONIA, TUN, and EVN [38,66,67,68,69,70]. FER (FERONIA) accumulates asymmetrically in the filiform apparatus, and its signaling network controls the release of sperm cells from the pollen tube [39,71,72,73]. This is related to an increase in ROS levels induced by FER at the micropylar region, leading to an elevated Ca^2+^ concentration in the distal cytoplasm of the pollen tube [40,74]. In the *fer* mutant, the synergid cell does not undergo normal degeneration, thus retaining its ability to attract multiple pollen tubes [41]. FER interacts with demethylesterified pectin through its malectin domain, which activates the ROP6 signaling pathway. This interaction allows FER to sense and/or transduce mechanical stress, reorganize cortical microtubules, and maintain cell wall integrity [42,75,76,77,78]. Upon the arrival of the first pollen tube, FER induces nitric oxide (NO), which modifies LURE, blocking its secretion and preventing LURE from binding to its receptor, thereby suppressing further pollen tube attraction [43].

FER and LORELEI-like glycosylphosphatidyl-inositol-anchor protein 1/LORELEI (LLG1/LRE) interact both at the endoplasmic reticulum (ER) and the cell membrane, serving as integral components of the RAC/ROP signaling complex and playing a role in the rupture of the pollen tube [56,79,80]. The LRE gene is expressed in synergid cells before fertilization and encodes a conserved plant-specific GPI-anchored protein [54]. The MYB-related REVEILLE transcription factor regulates LRE expression in synergid cells by binding to the SEEL motif (‘TAATATCT’) in the LRE promoter [81]. A modified eight-cysteine motif plays a crucial role in *LRE* function during pollen tube reception [55]. The interaction between pectin and RALF triggers the FER-LLG1 signaling pathway, inducing massive endocytosis as a stress response [82].

NTA (NORTIA) is a component of the FER signaling pathway that induces pollen tube rupture. In synergid cells, NTA-GFP accumulates at the micropylar end upon the arrival of the pollen tube, whereas in the *fer* mutant, no migration of NTA-GFP is observed [57,83]. The NTA (*At*MLO7) gene belongs to the plant-specific MILDEW RESISTANCE LOCUS O (MLO) protein family [84]. The accumulation of MLO proteins in Golgi-associated compartments is closely linked to their ability to complement the *nta-1* mutant phenotype [58]. Artificially fixing faNTA to the filiform apparatus significantly reduces the rates of unfertilized ovules caused by mutations in *fer* or *lre* by inhibiting pollen tube overgrowth [59]. The promotion of interspecific pollen tube acceptance by NTA and faNTA is FER-dependent [59,85]. Two additional *Cr*RLK1L homologs, HERK1 and ANJ, localize to the filiform apparatus and interact with FER-LRE to control NTA relocalization [45,86,87,88].

The FER-LRE complex in the filiform apparatus of the synergid cell receives signal molecules RALF4/19 released from the pollen tube, recruiting NTA to the filiform apparatus to form a Ca^2+^ channel, which induces Ca^2+^ influx and subsequent spikes, leading to the rupture of the pollen tube [57,59,60,89,90]. As Ca^2+^ concentrations rise, calmodulin (CaM) binds to NTA in a Ca^2+^-dependent manner, inhibiting NTA channel activity [60]. Additionally, ACC (1-aminocyclopropane-1-carboxylic acid) stimulates Ca^2+^ elevation through GLUTAMATE RECEPTOR-LIKE (GLR) channels in ovules, promoting the secretion of *At*LURE1.2 and attracting pollen tubes [49]. Specific *At*CNIHs (CORNICHON HOMOLOGS) regulate the sorting, localization, and trafficking of *At*GLRs, maintaining Ca^2+^ homeostasis [50]. The RALF N-terminal acts as a bridge in the heterodimerization of FER^ECD^ and LLG [91].

TUN and EVN play roles in the FER-mediated pollen tube acceptance pathway by participating in protein N-glycosylation in the endoplasmic reticulum [61]. Both mutants exhibit a *fer*-like phenotype with pollen tube overgrowth, and TUN may specifically be involved in the glycosylation of *Cr*RLK1L subfamily proteins [61,92]. Early nodulin-like proteins (ENODLs or ENs) are GPI-anchored proteins required for pollen tube bursting [46,93]. Specifically, EN14 interacts with the extracellular domain of FER, facilitating the polar anchoring of FER on the filiform apparatus [46].

The functional loss of AP1G and V-ATPases in synergids impairs vacuolar trafficking and acidification, leading to defective pollen tube reception and compromised synergid degeneration [62,94,95,96]. PICALM5 proteins, which contain the AP180 N-terminal homology (ANTH) domain, are involved in clathrin-mediated endocytosis occurring at the subapical plasma membrane of growing pollen tubes [97]. Through interaction with PICALM5A/B and PICALM9A/B, AP1G2 affects gametophyte cell division cycles, potentially influenced by Ca^2+^ dynamics [98].

*Zm*EA1, a hydrophobic 94-amino-acid small protein produced by egg apparatus cells with a transmembrane domain, plays a crucial role in maize short-range pollen tube guidance as a signaling molecule, attracting and arresting maize pollen tubes in vitro in a species-preferential manner to the micropylar opening of *Arabidopsis* ovules [27,28]. Defensin-like *Zm*ES4 (*Zea mays* embryo sac) targets K^+^ channels, including KZM1, to modulate sperm release after osmotic burst [29,99,100].

### 2.2. Central Cell Has an Impact on Pollen Tube Guidance

Some genes that influence the functional specialization of the central cell can affect pollen tube guidance when mutated, such as *CCG* and CBP1. The central cell can also secrete attractant peptides in cases where the synergid cell attraction fails. Loss of *CENTRAL CELL GUIDANCE* (*CCG*) (Figure 1) function impairs female control of micropylar pollen tube guidance, although it does not affect the development of these gametophytic cells or the filiform apparatus [17]. CCG-BINDING PROTEIN1 (CBP1) (Figure 1) connects transcription factors and RNA polymerase II machinery to regulate pollen tube attraction through its interaction with *CCG* and the mediator complex [18]. In *Arabidopsis*, the central cell secretes peptides SAL1 and SAL2 (Figure 1) to attract pollen tubes when the synergid-dependent attraction system fails or is disrupted by pollen tubes carrying non-viable sperm cells [19]. Loss of the synergid-independent attraction system, which includes the SAL peptides, significantly reduces the embryo sac’s ability to recover from fertilization failure, a process referred to as fertilization recovery [19,101].

In *Arabidopsis*, the ER-localized CYTOKININ INDEPENDENT 1 (CKI1) protein, a two-component sensor histidine kinase, is distributed to the central cell via nuclear migration, specifying central cells and restricting egg cell fate [102,103,104]. This process involves the downstream action of histidine phosphotransfer proteins 2, 3, 5, and response regulators 10, 12, 18 [105,106,107,108,109].

The polytubey block in *Arabidopsis* exhibits a ‘dual control’ nature, regulated jointly by the fertilized egg cell and central cell, specifically mediated by FIS-PRC2-induced H3K27me modification through the central cell pathway [20]. FIS-PRC2 is a polycomb repressive complex specific to the central cell and endosperm [21]. Fertilization signaling activates ETHYLENE INSENSITIVE 3 (EIN3), which disrupts EIN3 degradation by EIN3-BINDING F-BOX1 (EBF1) and EBF2, leading to the upregulation of SENESCENCE-ASSOCIATED GENE29 (SAG29/SWEET15), a sugar transporter, at the micropylar end of the ovule, blocking pollen tube attraction [110,111,112,113]. Subsequently, the persistent synergid cell fuses with the endosperm, induced by central cell fertilization, which dilutes synergid cytoplasm with pollen tube attractants to prevent polytubey [20,22]. FIS-PRC2 eliminates synergid nuclei during mitosis, while egg cell fertilization activates ethylene signaling to disorganize them [22,114]. Research indicates that it is not ethylene itself, but ethylene signaling components, including EIL1 and EIN3, that are necessary for the degradation of persistent synergids [115]. Mitogen-activated protein kinase 4 (MPK4) prevents the premature death of synergids and enables pollen tube reception by suppressing SUMM2-mediated ROS accumulation and immune responses, monitored through the phosphorylation of CALMODULIN-BINDING RECEPTOR-LIKE CYTOPLASMIC KINASE 3 (CRCK3) [47,116,117,118]. MPK4-GFP is detected in the nucleus of synergids, egg cells, and central cells after cellularization [47].

The MADS-box protein *AGAMOUS-LIKE80* (*AGL80*) may function as a heterodimer with AGL61 within the central cell, controlling central cell differentiation after polar nuclei fusion [119,120]. *AGL80* can repress *MYB98* expression in the central cell, and ectopic expression of *AGL80* in synergid cells can also lead to defects in pollen tube guidance [52,120].

### 2.3. Egg Cell Play a Vital Role in Polytubey Block and Right Fertilization

The egg cell plays a crucial role in guiding the pollen tube and preventing polytubey. During gamete interaction, EGG CELL1 (EC1) is exocytosed from vesicles within the egg cell to accelerate gamete fusion and prevent polytubey by activating sperm in *Arabidopsis* [23]. EC1 proteins also contribute to the preferential fertilization of the egg cell over the central cell [121]. The correct fusion of the sperm and egg cell triggers the egg cell to secrete two aspartic endopeptidases, ECS1 and ECS2, from its cortical network to the filiform apparatus of the synergid cell [25]. ECS1 and ECS2 specifically cleave LURE1, establishing a rapid polyspermy-blocking mechanism in *A. thaliana* [25]. Egg cell-specific *RWP-RK domain-containing* (*RKD*) factors control cell differentiation and are required for normal gametophytic development [122,123].

In *Arabidopsis*, GEX3 encodes a plasma membrane-localizing protein expressed in both the male gametophyte and the egg cell [26]. The presence of GEX3 in the egg cell is crucial for micropylar pollen tube guidance in female gametophytes. Overexpression of GEX3 leads to the formation of non-viable embryos [26]. In rice, both overexpression and loss of function of *Os*GEX3 impact pollen maturation. Overexpression enhances osmotic and oxidative stress tolerance by increasing ROS scavenging in rice seedlings [124].

The ubiquitously expressed gene DHQS (3-dehydroquinate synthase) influences pollen tube funicular guidance. Mutants with disrupted DHQS show defects in pollen tube funicular guidance. However, specific expression of DHQS in the female germ unit—comprising synergid cells, the central cell, and the egg cell—can rescue the funicular guidance defect observed in the *dhqs/+* mutant [48]. It is believed that funicular guidance cues are established at the stage FG7 [48].

## 3. Pollen Tube Plays a Vital Role in Male–Female Communication

### 3.1. Pollen Tube Receptors and Signal Transduction Pathways

Several factors influence pollen germination and pollen tube elongation during guidance (Table 2) [8,10,75,125,126,127,128,129]. Two receptor-like kinase (RLK) genes, LOST IN POLLEN TUBE GUIDANCE 1 (LIP1) and 2 (LIP2), are anchored to the membrane at the pollen tube tip via palmitoylation and play a role in perceiving *At*LURE1 for micropylar guidance. Simultaneous inactivation of LIP1 and LIP2 results in impaired pollen tube entry into the micropyle and a significant reduction in the attraction of pollen tubes toward *At*LURE1 [130,131]. Plasma-membrane-localized receptor-like kinases PRK6 and MALE DISCOVERER1 (MDIS1), MDIS1-INTERACTING RECEPTOR LIKE KINASE1 (MIK1) and MIK2 (MDIS1-MIK1/2), located at the pollen tube tip, act as receptors for *At*LURE1. PRK6 accumulates increasingly toward the exogenous *At*LURE1 before the pollen tube redirects [132]. LURE1 binds to the extracellular domains of MDIS1, MIK1, and MIK2, triggering MDIS1–MIK dimerization and MIK1 autophosphorylation [133]. In *mdis1* and *mik1 mik2* mutant pollen tubes, the response to LURE1 is less sensitive [133]. Additionally, transferring the MDIS1 gene from *A. thaliana* to its sister species, *Capsella rubella,* partially breaks down the reproductive isolation barrier [133]. PRK6 appears to be the primary receptor for *At*LURE1.

**Figure 2:**
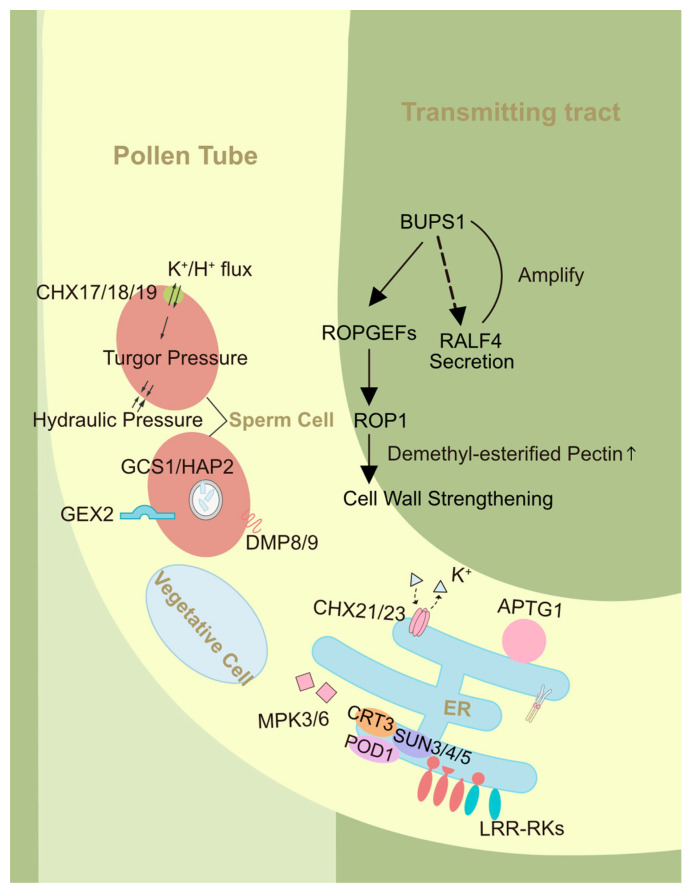
Pollen tube during growth. CHX21/23 located in the endoplasmic reticulum and CHX17/18/19 located in the sperm membrane are K^+^/H^+^ exchangers. SUN3/4/5-POD1-CRT3 regulates sorting of LRR-RKs to the plasma membrane. APTG1 affects the localization of GPI-anchored proteins such as COBL10. BUPS1 maintains the integrity of pollen tubes in the transmitting tract through a series of cascading reactions.

Wild-type (WT) ovules and *atlure1null* ovules show comparable micropylar attraction efficiency when pollinated with *prk6* pollen tubes, indicating that *At*LURE1/PRK6 signaling promotes the attraction of conspecific pollen tubes to the micropyle [163]. Unlike *lip1 lip2*, *mpk3 mpk6* (mitogen-activated protein kinases) (Figure 2) pollen tubes exhibit deficiencies in funicular guidance, while micropylar guidance remains normal [134]. ALA3 maintains the polar distribution of the specific anionic phosphatidylserine (PS) in pollen tube tips [164,178], and with that, PS regulates the localization and related vesicle trafficking of Rab GTPase RabA4d [164,179], which affects the polar transport of PRK6 to the pollen tube tip [164]. Then, the PRK6 receptor loses response to LURE, and pollen tubes in turn lose direction.

Phytosulfokine (PSK), expressed within the pollen, is activated by tyrosylprotein sulfotransferase (TPST) and transmits signals through its receptor PSKR, guiding the pollen tube from the transmitting tract to the funiculi [135]. ABNORMAL POLLEN TUBE GUIDANCE1 (APTG1) (Figure 2) is an ER-localized protein in the pollen tube and an *Arabidopsis* homolog of PIG-B and GPI10 [156]. In progeny derived from self-fertilization of the heterozygous *aptg1* mutant, the proportion of *aptg1* heterozygotes is significantly below 50% due to compromised pollen tube guidance, in accordance with Mendel’s laws of inheritance. Furthermore, embryos carrying homozygous aptg1 mutations exhibit lethality. Mutation of APTG1 also affects the localization of COBRA-LIKE10 (COBL10) (Figure 1), a GPI-anchored protein essential for pollen tube growth in the transmitting tract and micropylar guidance [156,165]. Mutation of COBL10 disrupts the deposition of the apical pectin cap and cellulose microfibrils, important structural components of the pollen tube wall [165].

### 3.2. Regulation of Pollen Tube Growth and Ion Homeostasis Involved in Pollen Tube Guidance

Some genes that contribute to pollen tube cell wall composition, growth, and the regulation of ion homeostasis also play a role in pollen tube guidance. Transcription factors *MYB97/101/120*, expressed in the pollen tube nucleus, play a role in pollen tube reception. Mutants in these factors exhibit pollen tubes that fail to stop growing in synergids and degenerate [160,161]. The receptor kinases FER, ANJ, and HERK1 are crucial for establishing the polytubey block, which relies on RALF6, 7, 16, 36, and 37 peptides from pollen tubes [39,45,139]. Successful fertilization leads to the programmed cell death of the synergid cell, degrading attractants and preventing polyspermy [22,25,139]. However, if fertilization fails, the synergid cell remains active, producing attractants and enabling secondary pollen tubes to emerge for salvage fertilization [139].

A novel ER luminal protein, POLLEN DEFECTIVE IN GUIDANCE1 (POD1), functions in retaining ER proteins and interacts with CALRETICULIN3 (CRT3), a luminal chaperone involved in Ca^2+^ homeostasis and ER quality control. This interaction is crucial for regulating pollen tube guidance [157]. Further research indicates that the SUN3/4/5-POD1-CRT3 (Figure 2) complex on the ER membrane ensures quality control of LRR-RKs, promoting their proper folding and sorting for anterograde trafficking [158,180].

Receptor-like kinases ANXUR1 (ANX1) and ANXUR2 (ANX2), localized on the pollen tube wall, prevent premature pollen tube rupture during growth. Interaction between ANX proteins and FER/SRE RLKs triggers pollen tube rupture [166,167]. The *MRI^R240C^* mutant, carrying an R240C amino acid substitution in the activation loop of MRI, partially rescues the tube-bursting phenotypes of *anx1 anx2*, suggesting that MRI functions downstream of the *Cr*RLK1L-dependent pathway during tip growth [181]. TUN appears to be essential for the glycosylation of ANX1 and ANX2, as ANX-GFP is undetectable in *tun* mutant pollen grains [61]. The *picalm5a picalm5b* double mutant severely impairs the tip localization of ANX1/2 proteins [97].

During pollen tube reception for the ovule, *Cr*RLK1L proteins BUPS1 and BUPS2 physically interact with ANX1/2 through their malectin domains, which also bind to RALF4 and RALF19 [89,166,167,182]. ANX-BUPS maintains pollen tube integrity in response to RALF4/19 signaling [89,183,184]. However, RALF34, which is highly expressed in mature ovules, especially around the micropyle and synergids, competitively binds to ANX-BUPS at the interface between the pollen tube and female gametophyte [89,185]. This competition disrupts signaling between BUPS and ANX, leading to pollen tube rupture and sperm release [89,184]. Down-regulation of RALF4/19 disrupts cell wall composition, causing bulges and pollen tube rupture due to acidic pectin accumulation, highlighting its role in regulating pollen tube integrity and growth via interactions with LRX proteins upstream of ANX1/2 [153,154]. Pollen-specific LLG2/3 serve as coreceptors of ANX/BUPS, promoting their secretion to the apical plasma membrane to perceive the RALF4 signal. This activates ROS production and stimulates pollen tube growth through a potential downstream ROP pathway [137,138]. BUPS1 (Figure 2) is crucial in maintaining the integrity of pollen tube cell walls by sensing and responding to mechanical stress, particularly as they traverse through the transmitting tract [65,168,186,187]. BUPS1 directly regulates ROP1 GTPase signaling, and its ligands, RALF4 and RALF19, amplify the mechanical signal, enabling the pollen tubes to adapt their cell wall rigidity in response to acute mechanical stress during penetrative growth [154,168,188].

GENERATIVE CELL SPECIFIC 1 (HAP2/GCS1) (Figure 2) has a carboxy-terminal transmembrane domain and is localized on the plasma membrane of generative cells [175,176]. The *gcs1* mutant exhibits defects in pollen tube guidance as well as gamete attachment and fusion [175,176]. In the knockdown of DOMAIN MEMBRANE PROTEIN 9 (DMP9), sperm cells fail to fuse with egg cells but fertilize the central cells instead [189,190]. HAP2/GCS1 interacts with two sperm-specific DOMAIN OF UNKNOWN FUNCTION 679 (DUF679) proteins, DMP8 and DMP9 (Figure 2), and co-localizes with them on the organelle-like structure of sperm cells [171]. Mutations in *dmp8/9* cause sperm cells to fail in eliciting a response to the EC1 peptide, resulting in defective relocalization of HAP2/GCS1-RFP from the endomembrane to the cell surface, ultimately leading to failure of sperm–egg fusion [172]. GEX2 (Figure 2), located within the sperm plasma membrane, is required for gamete adhesion, ensuring reliable double fertilization [173].

Changes in cell wall components also significantly affect pollen tube growth. PERK5 and PERK12 are proline-rich extensin-like receptor kinases that play redundant roles in pollen tube growth and control ROS homeostasis [145]. *perk5* and *perk12* mutants display excessive accumulation of pectin and cellulose on the cell wall of the pollen tube tip, resulting in increased wall hardness and shorter pollen tubes [145,191]. *Germinating modulator of rice pollen* (*GORI*) encodes a WD40 domain protein, the mutant pollen tubes exhibit reduced actin filaments and changes in pectin distribution. *GORI* plays a key role in endocytosis or exocytosis complexes that mediate rice pollen tube growth [147]. *Os*MTD2, *Cr*RLK1L male-gene transfer defective 2 in *Oryza sativa*, mediates ROS homeostasis and affects cell wall modification required for pollen tube integrity [148]. *Z. mays* pectin methylesterase inhibitor 1 (*Zm*PMEI1) likely destabilizes the pollen tube wall during perception and contributes to sperm release, along with other proteins such as *Zm*ES4 [149]. Leucine-rich repeat (LRR) extensins (LRXs) LRX8-11 proteins play crucial roles in pollen tube growth and cell wall formation [150,151,152]. They likely influence pollen tube growth and guidance by regulating cell wall composition, vesicle dynamics, Ca^2+^ signaling, and interactions with the plasma membrane [150,151,152]. Polygalacturonase *At*PGLR triggers cell wall remodeling, resulting in pollen tube elongation or rupture, which can be repaired by callose deposition [155].

During pollen tube growth, mitochondrial autophagy constantly occurs to provide energy [136,192,193,194]. SH3 DOMAIN-CONTAINING PROTEIN 2 (SH3P2) co-localizes with autophagy-related (ATG) proteins and mediates mitochondrial quality control to participate in this process [136,195]. LA and related protein 6C (LARP6C) regulate the translation, storage, and degradation of its mRNA targets through direct binding. Its absence disrupts lipid synthesis, homeostasis, and vesicular trafficking, thereby influencing pollen tube growth [196].

### 3.3. The Concentration Gradient of Ca^2+^ in Pollen Tubes Guidance

Calcium (Ca^2+^), hydrogen (H^+^), potassium (K^+^), and chloride (Cl^−^) are four key ions that regulate pollen tube growth [197,198] and also influence pollen tube guidance. Of these, Ca^2+^ plays a central role. It is well established that Ca^2+^ gradients at the tips of pollen tubes are critical for their guidance, with plasma membrane Ca^2+^ channels serving as essential regulators by facilitating external Ca^2+^ influx.

Plasma membrane Ca^2+^ channels at the pollen tube tips are core components that regulate Ca^2+^ gradients by mediating external Ca^2+^ influx. Specific Glutamate receptor-like channels (GLRs) located at the apical region of pollen tubes are activated by _D_-Serine, allowing Ca^2+^ to enter the cytoplasm [199]. Increased cytosolic calcium ion concentration ([Ca^2+^]_cyt_) activates S-type anion channels SLAH3 and ALMT-based R-type anion channels through Ca^2+^-dependent protein kinases (CPKs), which are crucial for normal pollen tube growth and morphology [200].

As the pollen tube approaches the ovule, attractants secreted by the synergid cells influence Ca^2+^ concentrations at the pollen tube tip, facilitating the interaction between the pollen tube and ovule [201].

Cyclic nucleotide-gated channels (CNGCs) are permeable to calcium ions and participate in environmental stimulus response, ion balance regulation, and developmental processes [202,203,204]. CNGC18 (Figure 1) localizes to the pollen tube tip membrane and is affected by tip-localized ROP1 signaling. It transduces cyclic nucleotide (cNMP) signals into calcium ion fluxes for polarized tip-growth regulation [205,206]. Calcium-activated CPK32 interacts with and activates CNGC18, further promoting calcium entry during the elevation phase of Ca^2+^ oscillations in pollen tube growth [207]. Point mutations R491Q and R578K in CNGC18 impair its activation by cNMPs, resulting in abnormal cytosolic Ca^2+^ gradients and severe defects in pollen tube guidance in *Arabidopsis* [169]. Calcium-dependent CaM2 regulates calcium influx channels by interacting with or dissociating from the CNGC18-CNGC8 heterotetramer, controlling cytosolic calcium levels [208]. MLO5, MLO9, and possibly MLO15 and MLO1 selectively recruit CNGC18 to the plasma membrane through the SNARE vesicle-associated membrane proteins VAMP721 and VAMP722 (Figure 1) [170,209]. RALF4/19 activate *At*MLOs calcium channels to maintain pollen tube integrity [210]. Receptor-like cytoplasmic kinases known as Delayed Burst (DEBs) localize on the plasma membrane and regulate the timely burst of pollen tubes by modulating Ca^2+^ dynamics via phosphorylation of P_2B_-ATPase ACA9 [140,141,142,143,144].

During pollen tube elongation, the vegetative cell precedes the two sperm cells, maintaining a fixed distance from the pollen tube tip. However, reduced Ca^2+^-dependent ROS-induced pollen tube bursts observed in *wit12* and *wifi* correlate with decreased proximity of the vegetative nucleus to the pollen tube tip [211,212,213,214]. During double fertilization, both the egg cell and central cell exhibit transient Ca^2+^ spikes, while the receptive and persistent synergid cells display distinct Ca^2+^ oscillation patterns [215,216].

K^+^ is abundant in pollen tubes and regulates several fundamental cellular processes, including turgor pressure and membrane potential during growth. The cation/proton exchanger CHX23, located in the ER of pollen tubes, mediates K^+^ transport, pollen tubes of *chx21 chx23* mutants become stalled in the transmitting tract and fail to reorient their growth direction to enter the funiculus [159]. In *chx17/18/19* mutants, sperm cells are spheroidal, fragile, and easily disintegrate within the embryo sac [174]. CPK11 phosphorylates CPK24 in a kinase cascade, mediating Ca^2+^-dependent inhibition of inward K^+^ channels in *Arabidopsis* pollen tubes, negatively regulating tube growth [217]. The receptor-like kinase *Ruptured Pollen tube* (*RUPO*) interacts with potassium transporters *Os*HAK1/19/20, and its phosphorylation/dephosphorylation regulates this interaction, crucial for potassium homeostasis and pollen tube growth and integrity [177]. Autoinhibited plasma membrane H^+^-ATPases (AHA) 6, AHA8, and AHA9 are indispensable for pollen tube growth and fertility. Their combined loss leads to defects in extracellular ion fluxes, plasma membrane potential, cytosolic pH gradients, actin organization, and ultimately premature pollen tube arrest [162,218].

## 4. Attraction in Moss, Ferns and Early Gymnosperm

In non-flowering plants, the female reproductive organ is the archegonium, with neck cells located at the opening. In early land plants such as mosses and ferns, sperm cells released into water can swim towards the archegonium with flagella, where they enter and fuse with the egg cell to complete fertilization. The structure of pollen tubes first emerged in Ginkgo and Cycad, but the ability for guidance developed later with the evolution of Pines and Cypresses [219,220]. In *Ginkgo* and Cycad, pollen grains are spread by the wind and captured by pollination drops [221], which absorb them. The grains then move a short distance to the nucellus and slowly form a pollen tube structure. After the archegonium matures, the pollen tube chamber releases sperm cells with multiple flagella, which swim into the archegonium through the neck cell [222,223,224,225,226,227].

Genes involved in archegonium genesis also affect sperm cell attraction. In *Marchantia polymorpha*, *Mp*CKI1 is essential for egg cell formation through two-component signaling mediated by histidine-containing phosphotransfer proteins and type-B response regulators [228,229,230]. *Mp*CKI1 regulates female germline specification by governing both the asymmetric distribution of *Mp*BONOBO (*Mp*BNB) during cell divisions and the maintenance of *Mp*BNB accumulation in the egg-cell lineage [228,231]. In *Ginkgo*, *Gb*CKI1 is strongly expressed in the central cell and neck cells during early archegonium development. It can partially rescue an *Arabidopsis cki1* mutant and promote weak activation of the cytokinin signaling pathway in the *Arabidopsis* embryo sac but does not confer central cell specification [232]. *MpRKD* is a key regulator of plant reproduction, controlling gametophyte development, egg cell quiescence, and preventing parthenogenesis to ensure proper fertilization and sporophyte development [233,234]. *M. polymorpha FEMALE GAMETOPHYTE MYB* (*MpFGMYB*) is a female-specific gene, and its expression is repressed by the *cis*-acting antisense gene *SUF* in males, ensuring sexual dimorphism [235,236,237,238]. The repression of *SUF* is mediated by its transcription into the 3ʹ overlapping region [239]. Disruption of either *MpFGMYB* or *SUF* results in sexual conversion, with *suf* mutants lacking egg cells and *Mpfgmyb* mutants producing sperm lacking motility [235,237]. Liverworts possess U or V sex chromosomes, associated with female and male gender, respectively [229,240,241]. The U-linked *Feminizer*, *BASIC PENTACYSTEINE ON THE U CHROMOSOME* (*BPCU*) represses *SUF* expression in females [242]. *BRI1-EMS-SUPPRESSOR 1/BRASSINAZOLE-RESISTANT 1* (*BES/BZR*) transcription factors are major regulators in the brassinosteroid signaling pathway, with *MpBES1* and *MpBZR3* belonging to this family [229]. *MpBES1* regulates cell division and differentiation in the meristem [243]. *MpBZR3* regulates early antheridium development and late egg maturation and maintenance during archegonium development, which is crucial for the function of archegonium [244].

There is also a scent-based ‘plant–pollinator-like’ relationship between mosses and microarthropods. Fertile moss shoots attract springtails and mites, which carry moss sperm, thereby enhancing the fertilization process [245]. Tissues of the cosmopolitan moss *Ceratodon purpureus* emit complex volatile scents, with the chemical composition being sex specific. Moss-dwelling microarthropods are differentially attracted to these sex-specific volatile cues, significantly increasing moss fertilization rates [246].

The emergence of R2R3 MYB transcription factor *DUO1* and its orthologs represents a pivotal evolutionary development, enabling sperm differentiation and diverse sexual reproduction modes in land plants [247,248]. *DUO1* functions specifically in the male germline of *Arabidopsis*, controlling mitosis to form sperm cells [249,250,251,252], and also plays a role in the elongation of the generative cell [253]. In mosses, GLUTAMATE RECEPTOR-LIKE channels also contribute to sperm cell guidance. Mutations in *glr1* and *glr2* in *Physcomitrella patens* result in sperm cells failing to target the entrance of archegonia, due to the role of GLR-mediated regulation of cytoplasmic Ca^2+^ [254]. The homeodomain protein BELL1 is a key regulator of the transition from gametophyte to sporophyte in *P. patens*, with loss-of-function mutants being unable to form embryos [255]. GLRs may regulate BELL1 expression upstream in *P. patens* [254].

## 5. Conclusions and Outlook

In summary, the process of pollen tube guidance in angiosperms represents a highly intricate and evolutionarily refined mechanism that ensures successful fertilization. This guidance involves complex signaling interactions between the female gametophyte and the male gametophyte, allowing precise and timely targeting of the ovule. The role of various female gametophyte cells, such as synergid cells, central cells, and egg cells, in attracting the pollen tube and regulating fertilization is critical to understanding reproductive success in angiosperms. Insights into the molecular and cellular dynamics of these interactions, particularly the signaling pathways and factors involved in pollen tube navigation, have expanded our knowledge of plant reproductive biology.

While significant progress has been made, many aspects of pollen tube guidance remain to be explored. Future research should focus on the detailed molecular mechanisms underlying pollen tube signaling and how these signals are integrated at the cellular level. The role of additional factors such as small RNAs, epigenetic modifications, and long-distance signals in regulating pollen tube growth and targeting could offer new insights. Moreover, understanding how environmental stress conditions, such as temperature fluctuations or water availability, impact pollen tube guidance and fertilization success could provide valuable knowledge for improving crop resilience.

Additionally, comparative studies between angiosperms and earlier-diverging plant lineages, such as gymnosperms and other archegoniate plants, could offer a broader perspective on the evolution of fertilization mechanisms. These insights may help identify conserved and divergent elements in pollen tube guidance across plant species, shedding light on the evolutionary pressures that shaped reproductive strategies in the plant kingdom.

In conclusion, continued research in pollen tube guidance offers promising potential not only for advancing fundamental plant biology but also for developing strategies to improve crop breeding [44], fertility, and adaptation to changing environments. A deeper understanding of these processes will undoubtedly play a critical role in addressing global agricultural challenges in the future.

## Figures and Tables

**Table 1:** Summary of female genes involved in pollen tube guidance.

Gene Name	Express	Properties	Function	References
CCG	CC	Encode a N-terminal zinc β-ribbon domain	Micropylar guidance	[17]
CBP1	CC	CCG-BINDING PROTEIN1	Micropylar guidance	[18]
SAL1 and SAL2	CC	Peptide attractants	Fertilization recovery	[19]
FIS-PRC2	CC and endosperm	Fertilization-independent seed-class Polycomb Repressive Complex 2	Mitosis-associated synergid nuclear elimination and polytubey block	[20,21,22]
EC1	EC	CRPs	Induce redistribution of HAP2/GCS1 to sperm membrane	[23,24]
ECS1 and ECS2	EC	Aspartic endopeptidases	Cleave LURE1 after successful fertilization	[25]
GEX3	EC and male gametophyte	Encode a N-terminal PQQ domain	Micropylar guidance	[26]
*Zm*EA1	Egg apparatus	Hydrophobic protein	Micropylar guidance	[27,28]
*Zm*ES4	embryo sac	DEFL CRPs	PT rupture and targets K^+^ channels KZM1	[29]
MYB98	FA	R2R3 MYB transcription factor	FA formation and micropylar guidance	[30,31,32]
LURE	FA	DEFL CRPs	Micropylar guidance	[33,34,35]
XIUQIU	FA	CRPs	Micropylar guidance	[36]
TICKET	FA	CRPs	Micropylar guidance	[37]
FER	FA	RLK	PT and polytubey block	[38,39,40,41,42,43,44]
HERK1 and ANJ	FA	RLK	Interact with FER-LRE	[45]
ENODLs	FA	Early nodulin-like and GPI-anchored protein	Interact with FER-LRE	[46]
MPK4	FG	Mitogen-activated protein kinase	Determines synergid cell death	[47]
DHQS	FG	3-dehydroquinate	Funiculus guidance	[48]
ACC	Ovular sporophytic tissue	1-aminocyclopropane-1-carboxylic acid	Stimulates transient Ca^2+^ elevation via GLR channels	[49,50]
AMOR	Ovary	Methyl-glucuronosyl arabinogalactan	Induce PT respond to LURE	[51]
AGL80	ovule	MADS-box family	Central cell and endosperm development	[52]
NPA1	SCs	Peptide attractants	Micropylar guidance	[53]
LRE	SCs	GPI-anchored protein	FER chaperone	[54,55,56]
NTA (*At*MLO7)	SCs	MILDEW RESISTANCE LOCUS O protein family	PT rupture	[57,58,59,60]
TUN and EVN	SCs	TUN encodes UDP-glycosyltransferase EVN encodes dolichol kinase	PT reception mediated by FER	[61]
AP1G	SCs	Tetrameric protein complex	Mediate vacuolar acidification	[62]

Note: FA: filiform apparatus. PT: pollen tube. SCs: synergid cells. CC: central cell. EC: egg cell. FG: female gametophyte. DEFL: defensin-like. CRPs: cysteine-rich proteins. RLK: receptor-like kinases.

**Table 2:** Summary of male genes involved in pollen tube guidance.

Gene Name	Express	Properties	Function	References
MPK3 and MPK6	Male	Mitogen-activated protein kinase	Funiculus guidance	[134]
PSK	Male	Phytosulfokine peptide	Funiculus guidance	[135]
SH3P2	PT	Non-ATG regulator of plant autophagy	Mediates mitochondrial quality control	[136]
RALF4 and RALF19	PT	Rapid alkalinization factor	Peptide ligands for the ANX-BUPS1	[89]
LLG2 and LLG3	PT	GPI-anchored protein	Facilitate the secretion of ANX-BUPS1 to PT tip	[137,138]
RALF6/7/16/36/37	PT	Rapid alkalinization factor	Interact with FER/HERK1/ANJ to establish polytubey block	[139]
ACA9	PT	Ca^2+^ pump P_2B_-ATPase	Interact with DEBs	[140,141,142,143,144]
DEBs	PT	Receptor-like cytoplasmic kinases	PT rupture	[144]
PERK5 and PERK12	PT	Proline-rich extensin-like receptor kinases	Control ROS homeostasis	[145,146]
GORI	PT	WD40 domain protein	PT growth	[147]
*Os*MTD2	PT	*Cr*RLK1L family	PT integrity	[148]
*Zm*PMEI1	PT	Pectin methylesterase inhibitor	Destabilizes the pollen tube wall	[149]
LRX8-11	PT	Leucine-rich repeat extensins proteins	Regulate cell wall composition and interact with RALF4/19	[150,151,152,153,154]
*At*PGLR	PT	Polygalacturonase	Triggers cell wall remodeling	[155]
APTG1	PT ER	Mannosyltransferase	Participate in biosynthesis of GPI anchors	[156]
POD1-SUN-CRT3	PT ER	Chaperone complex	ER sorting of LRR receptor kinases	[157,158]
CHX21 and CHX23	PT ER	Cation/proton exchangers	Mediates K^+^ transport and funiculus guidance	[159]
MYB97/101/120	PT nucleus	MYB transcription factors	PT rupture	[160,161]
AHAs	PT plasma membrane	H^+^ ATPases	Sustain the ionic circuit	[162]
LIP1 and LIP2	PT tip	Receptor-like cytoplasmic kinase	Perceive *At*LURE1 signal	[131]
PRK6	PT tip	Pollen-specific receptor-like kinase	Perceive exogenous *At*LURE1 signal by asymmetric accumulation	[132,163]
ALA3	PT tip	P4-ATPase	Regulate the polar localization of PRK3 and PRK6	[164]
MDIS1-MIK1/2	PT tip	LRR-RLK	LURE receptor	[133]
COBL10	PT tip	GPI-anchored protein	Mediate directional growth of PT	[165]
ANX-BUPS1	PT tip	RLK	Maintain PT integrity	[166,167,168]
CNGC18	PT tip	Ca^2+^ channel	Mediate Ca^2+^ influx	[169]
MLO5/MLO9/MLO15	PT tip	MILDEW RESISTANCE LOCUS O protein family	Recruit CNGC18 to the PT tip through VAMP	[170]
DMP8 and DMP9	Sperm	DOMAIN OF UNKNOWN FUNCTION 679 membrane proteins	Participate in EC1-induced sperm activation	[171,172]
GEX2	Sperm	Membrane protein	Gamete attachment	[173]
CHX17/18/19	Sperm endomembrane	Cation/proton exchangers	Regulate the osmotic pressure of sperm	[174]
GCS1/HAP2	Sperm membrane	Transmembrane protein	Gamete fusion	[175,176]
RUPO	The apical plasma membrane and vesicle of PT	*Cr*RLK1L family	Regulates potassium homeostasis	[177]

Note: ER: endoplasmic reticulum.

## Data Availability

No new data were created or analyzed in this study. Data sharing is not applicable to this article.
